# Epigallocatechin Gallate (EGCG), a Green Tea Polyphenol, Reduces Coronavirus Replication in a Mouse Model

**DOI:** 10.3390/v13122533

**Published:** 2021-12-17

**Authors:** Rackhyun Park, Minsu Jang, Yea-In Park, Yeonjeong Park, Woochul Jung, Jayhyun Park, Junsoo Park

**Affiliations:** 1 Division of Biological Science and Technology, Yonsei University, Wonju 26493, Korea; rockhyun@yonsei.ac.kr (R.P.); minsujang@yonsei.ac.kr (M.J.); pyi012324@yonsei.ac.kr (Y.-I.P.); bbling408@yonsei.ac.kr (Y.P.); 2 Department of Analysis and Assessment, Mine Reclamation Corporation, Wonju 26464, Korea; woochul@komir.or.kr (W.J.); jayhp@komir.or.kr (J.P.)

**Keywords:** coronavirus, green tea, EGCG, HCoV-OC43

## Abstract

The COVID-19 pandemic has resulted in a huge number of deaths from 2020 to 2021; however, effective antiviral drugs against SARS-CoV-2 are currently under development. Recent studies have demonstrated that green tea polyphenols, particularly EGCG, inhibit coronavirus enzymes as well as coronavirus replication in vitro. Herein, we examined the inhibitory effect of green tea polyphenols on coronavirus replication in a mouse model. We used epigallocatechin gallate (EGCG) and green tea polyphenols containing more than 60% catechin (GTP60) and human coronavirus OC43 (HCoV-OC43) as a surrogate for SARS-CoV-2. Scanning electron microscopy analysis results showed that HCoV-OC43 infection resulted in virion particle production in infected cells. EGCG and GTP60 treatment reduced coronavirus protein and virus production in the cells. Finally, EGCG- and GTP60-fed mice exhibited reduced levels of coronavirus RNA in mouse lungs. These results demonstrate that green tea polyphenol treatment is effective in decreasing the level of coronavirus in vivo.

## 1. Introduction

The COVID-19 pandemic has resulted in millions of deaths from 2020 to 2021 due to the high mortality of SARS-CoV-2 [1]. Although vaccines for SARS-CoV-2 are now available, new variants of SARS-CoV-2 are continuously emerging, and effective medicines for COVID-19 are under development [2]. Any medicine that can reduce the coronavirus in vivo will undoubtedly be helpful in improving the current COVID-19 conditions.

Green tea has been consumed for thousands of years, and many beneficial effects of green tea have been reported [3,4]. Recent studies have demonstrated that green tea and green tea polyphenols inhibit coronavirus proteins and coronavirus replication in vitro [5,6]. Many viruses, including coronaviruses, encode polyproteins, and viral or cellular proteases cleave polyproteins into functional individual proteins [7]. Therefore, virus-encoded proteases are regarded as major targets of antiviral medicines [8]. Coronavirus encodes two viral proteases, papain-like protease and chymotrypsin-like protease (3CL protease), which have more cleavage sites in coronavirus proteins [9]. Therefore, 3CL protease is the primary target of coronavirus drugs, and several reports have demonstrated that green tea polyphenols, including EGCG, inhibit coronavirus 3CL-protease [10,11,12,13]. Coronaviruses use the spike protein for entry into host cells, and the coronavirus spike protein–receptor interaction is known to be the target of green tea polyphenol [14]. Recent reports suggest that EGCG prevents the interaction between coronavirus spike protein and cellular receptors and inhibits the entry of coronavirus into host cells [15]. In addition, EGCG has been reported to inhibit NSP15 endoribonuclease activity in vitro [16]. These results collectively indicate that green tea polyphenols can inhibit coronavirus proteins.

Because green tea is a very popular beverage, there has been attempts to evaluate the correlation between average green tea consumption and COVID-19 morbidity/mortality, and countries with higher rates of green tea consumption showed less morbidity/mortality than those with lower green tea consumption [17]. Recently, small clinical studies have been performed to study the efficacy of green tea consumption in COVID-19 treatment, and the results are promising [18].

Coronaviruses are classified into alpha, beta, gamma, and delta coronaviruses, and only alpha and beta coronaviruses are reported to infect humans [19]. Seven coronavirus strains are known to infect humans: human coronavirus 229E (HCoV-229), HCoV-HKU1, HCoV-NL63, HCoV-OC43, SARS-CoV, MERS-CoV, and SARS-CoV-2 [20]. As SARS-CoV-2 and HCoV-OC43 belong to the beta coronavirus family, we used the HCoV-OC43 virus as a surrogate in this report. We demonstrated that green tea polyphenol extract and EGCG treatment can reduce coronavirus in mice. To the best of our knowledge, this is the first study to investigate the effect of green tea on coronavirus replication in a mouse model.

## 2. Materials and Methods

### 2.1. Cell Culture and Infection to Cells

RD and HCT8 cells were maintained in MEM (Welgene, Seoul, Korea) containing 10% fetal bovine serum (FBS, Thermo Fisher Scientific, Waltham, MA, USA) and 1% penicillin-streptomycin solution (Welgene). RD cells were infected with HCoV-OC43, as described previously [6]. The HCoV-OC43 virus was obtained from ATCC (Rockville, MD, USA). RD and HCT8 cells were obtained from the Korean Cell Line Bank (KCLB, Seoul, Korea). EGCG and GTP60 (polyphenon 60; polyphenols from green tea containing more than 60% catechins) were purchased from Sigma-Aldrich (St. Louis, MO, USA). GTP60 contained (-) epicatechin-3-gallate (21.0%), (-) epicatechin (7.3%), (-) epigallocatechin (7.9%), and (-) epigallo-catechin-3-gallate (29.2%) [21].

### 2.2. Mouse Experiment

Male C57BL/6 mice (3-week-old) were obtained from DBL (Seoul, Korea) and housed with wood chip bedding, clean-air rooms with a 12-h light–dark cycle, and a relative humidity of 50%. Mice were infected with 10 μL of HCoV-OC43 virus (10^7^ PFU/mL) through intranasal injection [22]. After infection, 30 mg/kg body weight GTP60 (Polyphenon 60) or 10 mg/kg body weight EGCG were administered daily for 2 weeks via regular drinking bottles to avoid stress exposure resulting from repeated injections [21,23,24]. Water bottles were replaced daily. After administration, mice were sacrificed by carbon dioxide (CO_2_) euthanasia, and samples were collected.

### 2.3. Quantitative RT-PCR and Western Blots

Quantitative RT-PCR was used to measure the level of coronavirus RNA in cells and media, as described previously [6]. Briefly, cells and media were harvested and RNA was extracted using Trizol (Thermo Fisher Scientific) in accordance with the manufacturer’s instructions and subjected to RT-PCR using the StepOnePlus Real-Time PCR System (Thermo Fisher Scientific). The HCoV-OC43 N gene was amplified using the forward primer 5′-AGG ATG CCA COCA AAC CTC AG-3′ and reverse primer 5′-TGG GGA ACT GTG GGT CAC TA-3′. Western blotting was used to measure the level of coronavirus protein in the cells, as described previously [6]. Briefly, coronavirus-infected HCT8 cells were harvested and resuspended in cell lysis buffer (150 mM NaCl, 50 mM HEPES (pH 7.5), and 1% NP40) containing a protease inhibitor cocktail (Roche, Basel, Switzerland). Equal amounts of proteins were subjected to Western blotting with anti-HCoV OC43 antibody (Sigma-Aldrich). Images were acquired using the ImageQuant LAS 4000 system (GE Healthcare, Waukesha, WI, USA).

### 2.4. Scanning Electron Microscopy

For SEM imaging, HCT8 cells were grown on sterilized 9 mm cover slips and infected with HCoV-OC43 for 3 days. Cells were fixed with 2.5% glutaraldehyde for 1 h and immediately dehydrated in an ethanol series (20%, 40%, 60%, 80%, 90%, and 100% Et-OH). After dehydration, the cells were air-dried using a vacuum desiccator. Cells were coated with platinum, and images were captured using a Carl Zeiss SEM SUPRA 40 microscope (Carl Zeiss, Oberkochen, Germany).

### 2.5. Statistical Analysis

The results of Western blotting and quantitative RT-PCR were evaluated by a 2-tailed Student’s *t*-test using Excel software, Excel 2016 (Microsoft, Redmond, WA, USA). Statistical significance was set at *p* < 0.05.

## 3. Results

### 3.1. Coronavirus Infection Results in Cell Surface Alteration

**Figure 1:**
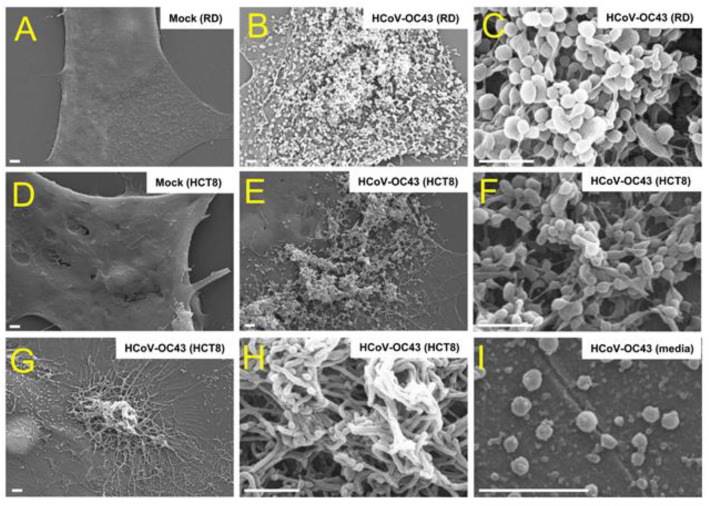
Scanning electron microscope (SEM) images of coronavirus-infected cells. RD cells and HCT8 cells were infected with mock or HCoV-OC43 (10^6^ PFU/mL). Briefly, 72 h after infection, cells were processed and analyzed via SEM. Mock-infected cells showed smooth surfaces (**A**,**D**), and HCoV-OC43-infected cells showed virus particles in RD cells (**B**,**C**) and HCT8 cells (**E**,**F**). HCoV-OC43-infected HCT8 cells showed long surface projections (**G**,**H**). HCoV-OC43 virion particles in media were analyzed (**I**). Scale bars, 1 μm.

To examine the coronavirus infection on the surface, we infected RD and HCT8 cells with HCoV-OC43 strain. Three days after infection, we used a scanning electron microscope (SEM) to analyze the infected cells. The mock-infected control cells showed a smooth surface, and coronavirus infection resulted in coronavirus virion particles on the cell surface (Figure 1A–F). We also analyzed the coronavirus virion particles in the media and found that the size of the coronavirus particles was approximately 100 nm (Figure 1I). Furthermore, we found that coronavirus infection of HCT8 cells produced extended surface projections (Figure 1G,H). These results indicate that coronavirus infection results in virion particle production on the surface, as well as cell surface projections.

### 3.2. EGCG and Green Tea Polyphenols Inhibits Coronavirus Replication

**Figure 2:**
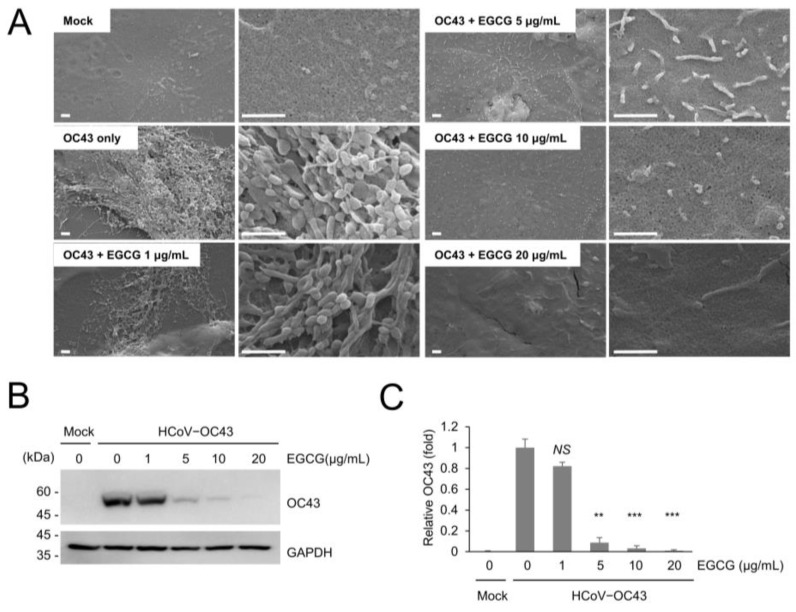
EGCG treatment inhibiting the coronavirus replication in HCT8 cells. (**A**) EGCG treatment decreases cell surface projections in HCT8 cells. HCT8 cells were infected with HCoV-OC43 and treated with the indicated concentration of EGCG. Briefly, 72 h after infection, cells were analyzed via SEM. Scale bars, 1 μm. (**B**) HCT8 cells were infected with HCoV-OC43, and HCoV-OC43-infected HCT8 cells were analyzed by Western blot with anti-OC43 antibody after 4 d of infection. (**C**) Level of OC43 bands was quantified by Western blot. The experiments were performed in triplicate, and the graph shows mean and standard error. Control vs. EGCG treatment: **: *p* < 0.01, ***: *p* < 0.005, *NS*: not significant.

We examined the effect of green tea polyphenols on coronavirus replication in mice and produced the coronavirus in RD and HCT8 cells. As HCoV-OC43 virus produced in HCT8 cells results in efficient infection in mice, we performed the experiments with the coronavirus produced in HCT8 cells. We examined the inhibitory effect of EGCG on coronavirus infection in HCT8 cells and found that EGCG treatment effectively decreased virus production and surface projection in HCT8 cells (Figure 2A). In addition, we analyzed HCoV-OC43 protein expression in HCT8 cells and found that EGCG decreased OC43 protein expression in a dose-dependent manner (Figure 2B). OC43 protein levels were significantly decreased after treatment with 5 μg/mL EGCG (Figure 2C).

**Figure 3:**
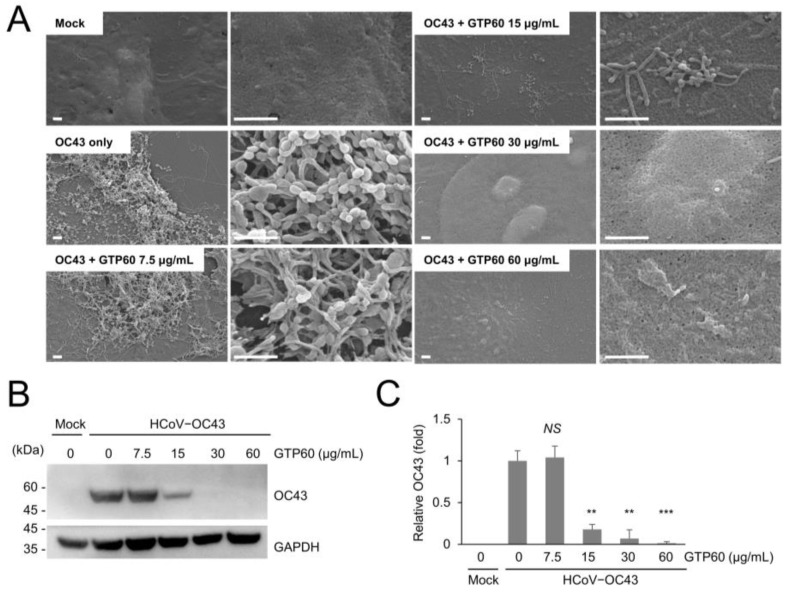
GTP60 treatment inhibiting the coronavirus replication in HCT8 cells. (**A**) GTP60 treatment decreases coronavirus-induced surface projections in HCT8 cells. Scale bars, 1 μm. (**B**) HCoV-OC43-infected HCT8 cells were analyzed by Western blot with anti-OC43 antibody. (**C**) Level of OC43 bands was quantified by Western blot. The experiments were performed in triplicate, and the graph shows mean and standard error. Control vs. GTP60 treatment, **: *p* < 0.01, ***: *p* < 0.005, *NS*: not significant.

Next, we examined the effects of green tea polyphenols on coronavirus replication. We used green tea polyphenols containing more than 60% catechin (GTP60), and SEM analysis demonstrated that GTP60 treatment decreased coronavirus-induced virus particle production and surface projections in HCT8 cells (Figure 3A) as well as coronavirus replication (Figure 3B). We found that treatment with more than 15 μg/mL GTP60 efficiently decreased HCoV-OC43 replication (Figure 3C).

**Figure 4:**
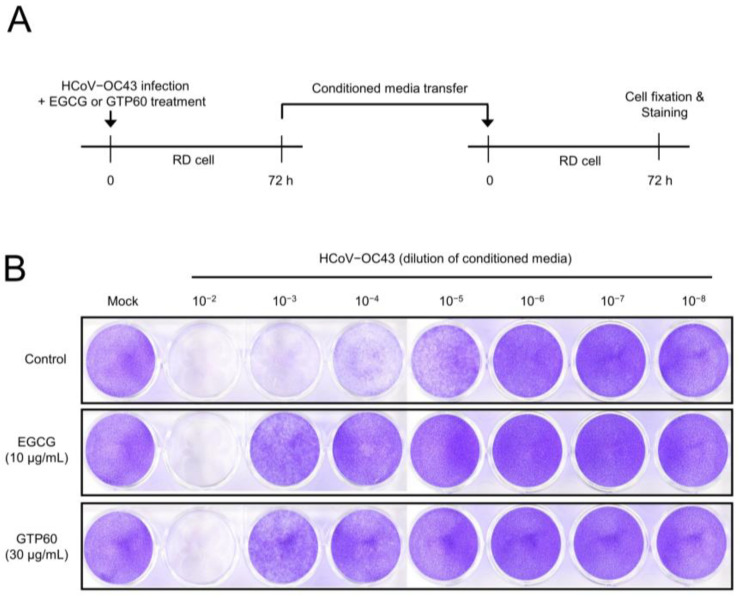
EGCG or GTP60 treatment reduced the infectivity of coronavirus. (**A**) Schematic diagram of experiment design. (**B**) RD cells were infected with HCoV-OC43 and treated with EGCG or GTP60. Briefly, 72 h after infection, the conditioned media were collected and transferred into uninfected cells. To visualize the cytotoxicity by infection, cells were fixed and stained with crystal violet after 72 h.

We also confirmed the reduction in coronavirus replication by examining coronavirus infectivity upon EGCG or GTP60 treatment. RD cells were infected with HCoV-OC43 and treated with EGCG or GTP60. After the media change, the infected cells were incubated for 72 h, and the conditioned media was used to infect uninfected cells (Figure 4A). Coronavirus infectivity was visualized by cytotoxicity, and the conditioned media from EGCG or GTP60-treated cells showed the reduced level of cytotoxicity (Figure 4B). These results indicate that EGCG or GTP60 treatment decreased the coronavirus replication and infectivity.

### 3.3. EGCG and Green Tea Polyphenols Reduce Coronavirus Replication in Mouse

**Figure 5:**
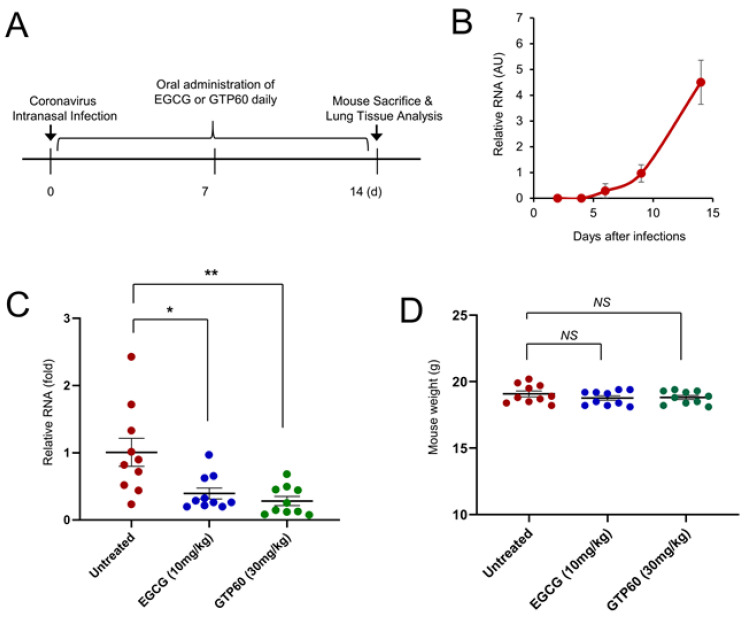
EGCG or GTP60-fed mice showing the reduced level of coronavirus RNA. (**A**) Schematic of mouse infection experiment design. Three-week-old mice were infected with HCoV-OC43 virus (10^5^ PFU) by intranasal infection, and the infected mice were left untreated or daily treated with EGCG, or GTP60. Two weeks after infection, mice were sacrificed, and coronavirus RNA was analyzed by quantitative RT-PCR. (**B**) Mouse were infected with HCoV-OC43 virus, and the expression of coronavirus RNA in the lung tissue was analyzed on the indicated day after infection. The graph shows mean and standard error. (**C**) EGCG and GTP60 decrease the level of coronavirus in mice. Ten mice in each group were used for HCoV-OC43 infection and treated with the indicated EGCG or GTP60 daily. After 2 weeks, the expression of virus RNA was evaluated through quantitative RT-PCR. The graph shows mean and standard error. Untreated vs. treatment, *: *p* < 0.05, **: *p* < 0.01 (n = 10). (**D**) After mouse sacrifice, the individual mouse weight was measured, and the data was depicted as a graph. Untreated vs. treatment, *NS*: not significant.

After we showed the inhibitory effect of EGCG and green tea polyphenols on coronavirus replication in vitro, we performed a mouse experiment to examine their inhibitory effect in vivo. Mice were infected with HCoV-OC43 virus, which was produced in HCT8 cells by intranasal infection, and the coronavirus RNA was evaluated by quantitative RT-PCR. We found that coronavirus RNA was readily detected in the mouse lung after two weeks (Figure 5A,B). However, we could not find any significant weight difference between the uninfected group and infected group (data not shown). To evaluate the effect of EGCG and GTP60, ten mice in each group were fed with untreated, EGCG (10 mg/kg), or GTP60 (30 mg/kg) daily for 2 weeks (Figure 5A). After sacrificing the mice, we examined the level of coronavirus in the lungs by quantitative RT-PCR. Compared with untreated mice, EGCG- or GTP60-fed mice showed reduced levels of coronavirus RNA in the lungs (Figure 5C). In addition, we measured the mouse weight in each group, and EGCG or GTP60 treatment did not result in a significant change in mouse weight, suggesting that EGCG or GTP60 treatment did not induce toxicity in mice (Figure 5D). These results collectively indicate that EGCG and GTP60 treatments are effective in inhibiting coronavirus replication in vivo.

## 4. Discussion

Mounting evidence indicates that green tea polyphenols such as EGCG inhibit coronavirus replication in vitro; however, experiments using mouse models have not yet been performed [5]. To the best of our knowledge, this report is the first to show that green tea polyphenols are effective in inhibiting coronavirus replication in vivo.

In this experiment, we used two types of compounds as a source of green tea polyphenols. One was EGCG, a major green tea polyphenol, and the other was a green tea extract from lab reagent suppliers (Sigma-Aldrich). Because the composition of green tea extract can be diverse based on the extraction methods, we used a standard green tea extract. Therefore, a similar experiment can be repeated by other researchers. In addition, we used the HCoV-OC43 virus for mouse experiments as a surrogate for SARS-CoV-2. Due to strict regulations, it is difficult to perform experiments with SARS-CoV-2. However, both SARS-CoV-2 and HCoV-OC43 are beta coronaviruses, and HCoV-OC43 is a good alternative for SARS-CoV-2 [20]. Further animal experiments will be required to examine the effect of green tea on SARS-CoV-2 replication.

Initially, we attempted to infect mice with HCoV-OC43 virus, which was produced in human RD cells; however, successful infection was not obtained with repeated trials. For this reason, we tested several other cell lines and found that the human HCT8 cell line was suitable for the production of HCoV-OC43 virus for infecting mice. Although we detected the replication of HCoV-OC43 in mice lungs, we did not observe any mouse death due to coronavirus infection, and we did not observe weight loss due to infection (data not shown). The effect of HCoV-OC43 infection on mice was mild and we did not observe any other external differences between the uninfected group and infected group. This is probably due to the pathological differences between humans and mice, and it is the limitation of animal experiments. To validate the effect of EGCG or green tea on coronavirus, further human clinical study should be conducted.

In this experiment, we evaluated the mouse intake of EGCG and GTP60 based on daily water consumption and reagent concentration [21]. As the intake amount of EGCG or GTP60 was reasonable, a man weighing 60 kg could obtain an equal amount of EGCG by consuming 600 mg of EGCG. Because green tea has been consumed for thousands of years, its safety is guaranteed. Therefore, green tea can be employed to reduce coronavirus in infected patients if the efficacy of green tea on coronavirus is thoroughly proven.

There are many reports supporting the efficacy of green tea against coronavirus. Green tea polyphenols, including EGCG, have been reported to inhibit several coronavirus proteins [5]. Moreover, green tea polyphenols have been shown to inhibit coronavirus replication, including SARS-CoV-2 [6,16]. In addition, a preliminary epidemiological study and small-scale clinical study suggest that the consumption of green tea can be beneficial for patients with COVID-19 [17,18]. Further preclinical and clinical trials should be conducted to clarify the efficacy of green tea against coronavirus disease.

## Data Availability

Not applicable.
